# Fungal bioremediation of soil co-contaminated with petroleum hydrocarbons and toxic metals

**DOI:** 10.1007/s00253-020-10854-y

**Published:** 2020-09-17

**Authors:** Qianwei Li, Jicheng Liu, Geoffrey Michael Gadd

**Affiliations:** 1 State Key Laboratory of Heavy Oil Processing, State Key Laboratory of Petroleum Pollution Control, China University of Petroleum-Beijing, Beijing, 102249 China; 2 Geomicrobiology Group, School of Life Sciences, University of Dundee, Dundee, Scotland DD1 5EH UK

**Keywords:** Fungi, Bioremediation, Petroleum hydrocarbons, Toxic metals

## Abstract

**Abstract:**

Much research has been carried out on the bacterial bioremediation of soil contaminated with petroleum hydrocarbons and toxic metals but much less is known about the potential of fungi in sites that are co-contaminated with both classes of pollutants. This article documents the roles of fungi in soil polluted with both petroleum hydrocarbons and toxic metals as well as the mechanisms involved in the biotransformation of such substances. Soil characteristics (e.g., structural components, pH, and temperature) and intracellular or excreted extracellular enzymes and metabolites are crucial factors which affect the efficiency of combined pollutant transformations. At present, bioremediation of soil co-contaminated with petroleum hydrocarbons and toxic metals is mostly focused on the removal, detoxification, or degradation efficiency of single or composite pollutants of each type. Little research has been carried out on the metabolism of fungi in response to complex pollutant stress. To overcome current bottlenecks in understanding fungal bioremediation, the potential of new approaches, e.g., gradient diffusion film technology (DGT) and metabolomics, is also discussed.

**Key points:**

• *Fungi play important roles in soil co-contaminated with TPH and toxic metals.*

• *Soil characteristics, enzymes, and metabolites are major factors in bioremediation.*

• *DGT and metabolomics can be applied to overcome current bottlenecks.*

## Co-contamination in the soil environment

With the accelerating pace of industrialization and urbanization, soil contamination has become a critical worldwide concern because of the threat to natural ecosystems and human health and much research has been carried out on innovative and cost-effective remediation technologies (Dong et al. 2013; Khan et al. 2018; Song et al. 2017). Soils co-contaminated with petroleum hydrocarbons and toxic metals are one of the major challenging problems in petroleum-producing countries, such as Qatar (Freije 2015), China (Cheng et al. 2019; Dong et al. 2013), and Russia (Kuyukina et al. 2018). Petroleum hydrocarbons and associated by-products found in soil are usually generated from accidental spills of crude oil, fuel contamination, refining processes, and subsequent problems associated with distribution and utilization. Spilled contaminants penetrate into soil pores and adsorb onto soil particles, moving vertically with capillary and gravitational forces which alters chemical, physical and biological properties, and composition (Czarny et al. 2020; dos Santos and Maranho 2018). Alkanes, aromatic compounds, nitrogen-sulfur-oxygen-containing compounds, and asphaltene are the major constituents of total petroleum hydrocarbons (TPH). The aromatic fraction refers to those compounds with benzene rings including polycyclic aromatic hydrocarbons (PAHs), which contain multiple fused aromatic rings, and are listed as priority pollutants due to their carcinogenic, mutagenic, and toxic properties as well as environmental recalcitrance (Czarny et al. 2020; Khan et al. 2018).

Toxic metals found in petroleum-contaminated soils include As, Ba, Cd, Cr, Pb, Hg, Ni, V, and Zn, and these are mainly associated with petroleum extraction and refining, and combustion of fuel for heat and transport (Adeniyi and Afolabi 2002; Klimek et al. 2016; Kuyukina et al. 2018; Muniz et al. 2004). The heavy oils in Russia are enriched with V and Ni, and trace amounts of Cd, Pb, and Zn (Kuyukina et al. 2018). More than 20 soil samples collected from petroleum-producing sites in China contained Cd, Ni, Cr, and Zn at concentrations ranging from 0.08–8.18, 21.6–40.7, 25.9–71.5, and 36.7–226.0 mg/kg dry weight, respectively (Cheng et al. 2019).

Remediation of soil co-contaminated with organic and inorganic pollutants is a complex problem as these two pollutant classes need to be treated differently (Gadd 2004). The presence of toxic metals in co-contaminated soil can inhibit petroleum biodegrading microorganisms, affecting growth and metabolism, nitrogen and sulfur conversions, and dehalogenation (Biswas et al. 2015; El-Azeem et al. 2013; Sandrin and Maier 2003). Toxic metal species may exert a plethora of toxic effects depending on metal concentration and speciation, physicochemical factors, and the organism’s ability to respond to metal stress through intrinsic or induced mechanisms (Gadd 1993; Gadd 2007; Rangel et al. 2018). Metals can exert toxicity in many ways, e.g., inhibition of enzymes, displacement or substitution of essential metals, disruption of cell and organellar membranes, and interaction with normal cellular homeostatic and stress response systems (Gadd 1993; Gadd 2007; Sullivan and Gadd 2019). For example, toxic metal cations may substitute for essential metal co-factors within an enzyme (e.g., Cd^2+^ may substitute for Zn^2+^) resulting in enzyme dysfunction (Sandrin and Maier 2003). Petroleum hydrocarbons in toxic metal-contaminated soils are hydrophobic materials with low water solubility and preferentially attach to the soil matrix which reduces the bioavailability of toxic metals to potential bioremediating microorganisms (Lai et al. 2009). Although research has been carried out on the bioremediation of co-contaminated soil with bacterial systems, much less attention has been paid to the potential roles of fungi in soils contaminated with petroleum hydrocarbons and toxic metals.

## Fungi in soil co-contaminated with petroleum hydrocarbons and toxic metals

Fungi are ubiquitous chemoorganotrophic (heterotrophic) organisms (Gadd 2008; Gadd 2017), and are one of the three major clades of eukaryotic life that independently evolved multicellular organization (Stajich et al. 2010). The colonization of soil by fungal mycelium results in enmeshment and aggregation of soil particles and improvement of soil structure, sometimes facilitating contaminant bioavailability (Harms et al. 2011). Compared with bacteria, filamentous fungi show some advantages in the transport or translocation of essential substances, including nutrients and water, and the pollutant itself, over significant distances (Boswell et al. 2003; Furuno et al. 2012; Boswell et al. 2002; Harms et al. 2011; Jacobs et al. 2002; Worrich et al. 2018). It is also significant that fungal mycelia can act as “highways” in facilitating the transport of pollutant-degrading bacteria over distance in soil which can enhance bioremediation (Banitz et al. 2013; Kohlmeier et al. 2005; Wick et al. 2007).

**Fig. 1:**
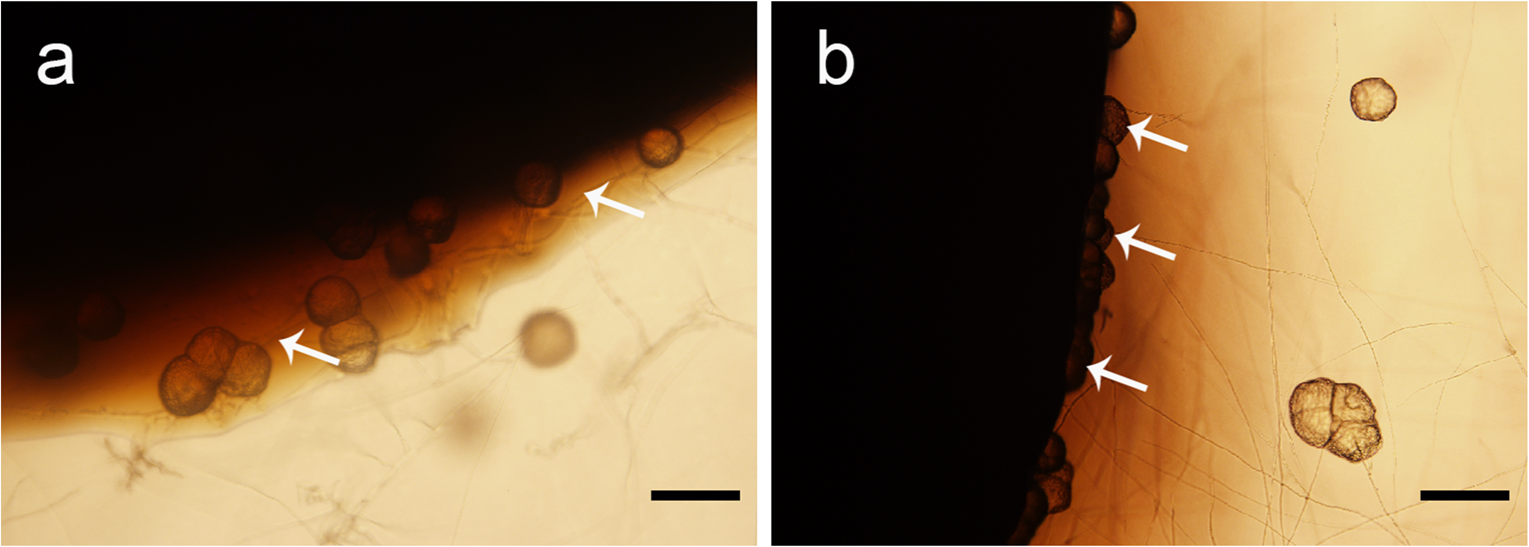
Fungal biomineralization of carbonates in media amended with heavy oil. *Neurospora crassa* was incubated on solid agar medium, supplemented with 40-mM urea and 50-mM CaCl_2_, at 25 °C in the dark for 5 days. Four wells (5-mm diameter) were made in the agar medium using a sterile cork borer and filled with heavy oil prior to fungal inoculation. Scale bars = 200 μm (Li et al., unpublished data)

**Fig. 2:**
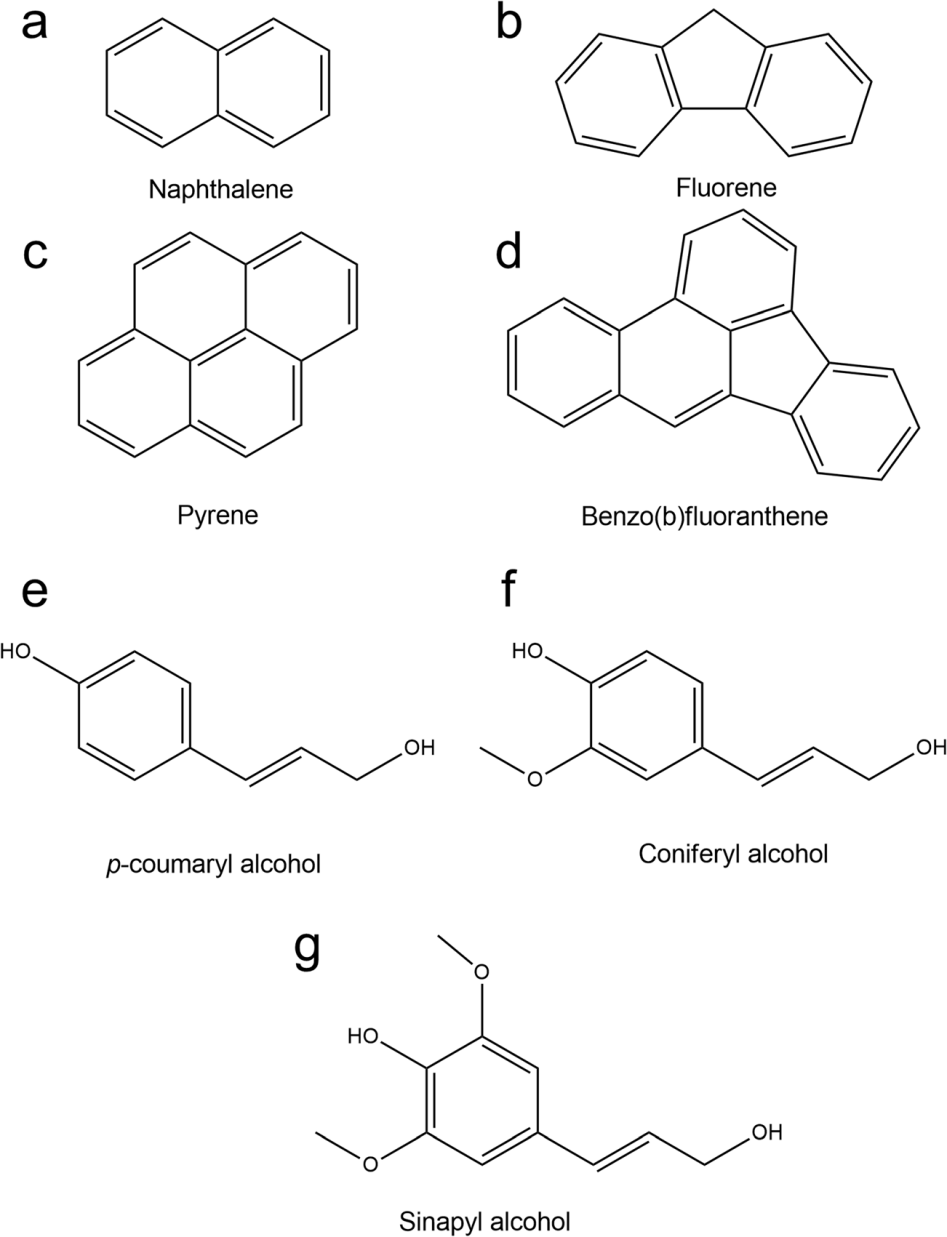
Chemical structure of some commonly studied PAHs and three constitutive monomers of lignin. PAHs are aromatic hydrocarbons with two or more fused benzene rings. **a** Naphthalene. **b** Fluorene. **c** Pyrene. **d** Benzo(b)fluoranthene. Lignin results from the enzymatic copolymerization of three phenolic monomers (monolignols): **e** *p*-coumaryl alcohol, **f** coniferyl alcohol, and **g** sinapyl alcohol

Many fungi can survive and grow in the presence of toxic metals and this depends on intrinsic biochemical and structural properties, physiological and/or genetic adaptation, including morphological changes, and environmental modification of metal speciation, bioavailability, and toxicity (Gadd 1993; Gadd 2010; Glasauer et al. 2004; Sullivan and Gadd 2019). Filamentous fungi, e.g., *Aspergillus* and *Penicillium* spp., have been investigated for the degradation of aliphatic hydrocarbons, chlorophenols, and polycyclic aromatic hydrocarbons, with the organic pollutants serving as carbon and energy sources (Harms et al. 2011; Hofrichter et al. 1994; Pinedo-Rivilla et al. 2009) (Table 1). The ability of ureolytic fungi, such as *Neurospora crassa*, to immobilize metals has been investigated because, when incubated in urea-supplemented media, toxic metals are precipitated as carbonates and/or oxides (Li et al. 2015; Li and Gadd 2017a; Li and Gadd 2017b; Li et al. 2019; Li et al. 2016; Li et al. 2014). When grown in urea-containing media supplemented with heavy oil and Ca^2+^, such mineral precipitation tended to aggregate along the edge of the heavy oil which may provide an additional carbon or energy source during the biomineralization process (Fig. 1). Moreover, fungi are primary decomposers of organic matter and plant biomass in soil with the chemical structure of lignin polymers of wood being comparable with the aromatic structure of PAHs (Haritash and Kaushik 2009; Vanholme et al. 2010) (Fig. 2). Because of this, many lignin-degrading fungi, e.g., *Phanerochaete chrysosporium*, have been investigated for degradation of PAHs and other aromatic compounds because of the wide range of substances that can be attacked by such organisms (Gadd 2004; Gadd 2001). Some fungi convert high-molecular-mass PAHs such as the highly carcinogenic benzo[α]pyrene into water-soluble products using non-specific detoxification mechanisms (Harms et al. 2011). *Fusarium solani* and *Hypocrea lixii* isolated from petrol station soil were investigated for the degradation of pyrene and tolerance to copper and zinc. These organisms degraded more than 60% of the supplied pyrene and could also accumulate Cu and Zn (Hong et al. 2010). In co-contaminated soil, Fe(III) coordinating fungal siderophores could play an important role not only by binding metals other than Fe(III), e.g., Cd, Cu, Ni, Pb, Zn, Th (IV), U(IV), and Pu (IV) (Ahmed and Holmström 2014) but also by facilitating the biodegradation of petroleum hydrocarbons by satisfying the Fe requirement for the degrading microorganisms in Fe-limited habitats. In co-contaminated soil, PAHs may interact with lipophilic components of the fungal cytoplasmic membrane, changing the permeability, which can result in penetration of toxic metals into cells and resulting effects/on cellular functions. Shen et al. (2005) investigated the effects of Cd and phenanthrene (Phe) on the growth of certain soil fungi and showed that growth was strongly inhibited in soil containing Cd and Phe compared with soil containing only Cd. Examples of selected fungal species interacting with PAHs and toxic metals are presented in Table 2.

**Table 1:**
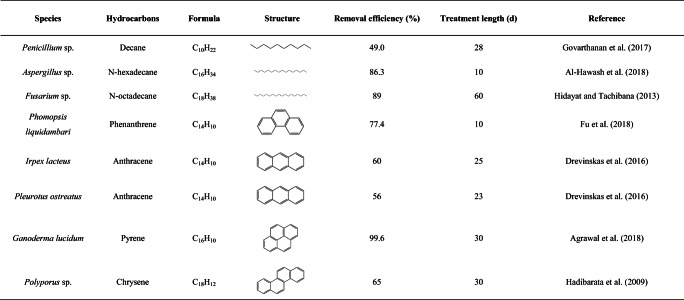
Some examples of degradation of petroleum hydrocarbons by different fungal species

**Table 2:** Some applications of fungal species for the bioremediation of PAHs and toxic metals

Fungus	PAHs	Initial content	Removal efficiency	Toxic metals	Initial content	Bioremediation efficiency	Data source	Reference
*Acremonium* sp.	Naphthalene, fluorine, phenanthrene, anthracene, fluoranthene	25 mg L^−1^	64.9–96.9%	Mn, Fe, Zn, Cu, Al, Pb	50 mg L^−1^	–	Lab	Ma et al. (2014)
*Pleurotus ostreatus*	Pyrene, benzo[a]anthracene, chrysene, benzo[k]fluoranthene, benzo[a]pyrene, dibenzo[a,h]anthracene, benzo[ghi]perylene	10 ppm	0.5–52.2%	Cd, Hg	10–500 ppm	–	Lab	Baldrian et al. (2000)
*Fusarium flocciferum*, *Trichoderma* sp., *Trametes versicolor*, *Pleurotus* sp.	Benzo[a]anthracene, benzo[a]fluoranthene, benzo[a]pyrene, chrysene, phenanthrene	150–250 mg kg^−1^	21–93%	Cd, Ni	50–500 mg kg^−1^	–	Lab	Al-Turki (2009)
*Pleurotus ostreatus*	Crude oil	–	–	Pb, Cu, Mn, Cd, Ni	0.79–52.07 mg kg^−1^	28.2–75.9%	Lab	Anacletus et al. (2017)

−, data unavailable

## Factors affecting the efficiency of fungal bioremediation

### Soil characteristics

Soil components, pH, and temperature are key factors in fungal bioremediation and metal speciation and affect the transportation and bioavailability of contaminants (Liu et al. 2017; Rangel et al. 2018). Organic matter and clay minerals significantly reduce the solution-phase concentration of metal ions. It was reported that in mineral-dominated soil, 0.01-mg L^−1^ Cd^2+^ inhibited the dechlorination of trichloroaniline (TCA) while 0.2-mg L^−1^ Cd^2+^ was necessary in an organic-dominated soil, which correlated with the metal-binding capacity of the organic material (Zhang et al. 2016). Clay minerals, e.g., montmorillonite, possess high cation exchange capacities (CECs), and can efficiently reduce metal bioavailability and toxicity (Sandrin and Maier 2003). Moreover, metals in soil may react with the organic pollutants to affect the speciation, bioavailability, and toxicity of the metal and the organic pollutant (Ceci et al. 2019).

**Fig. 3:**
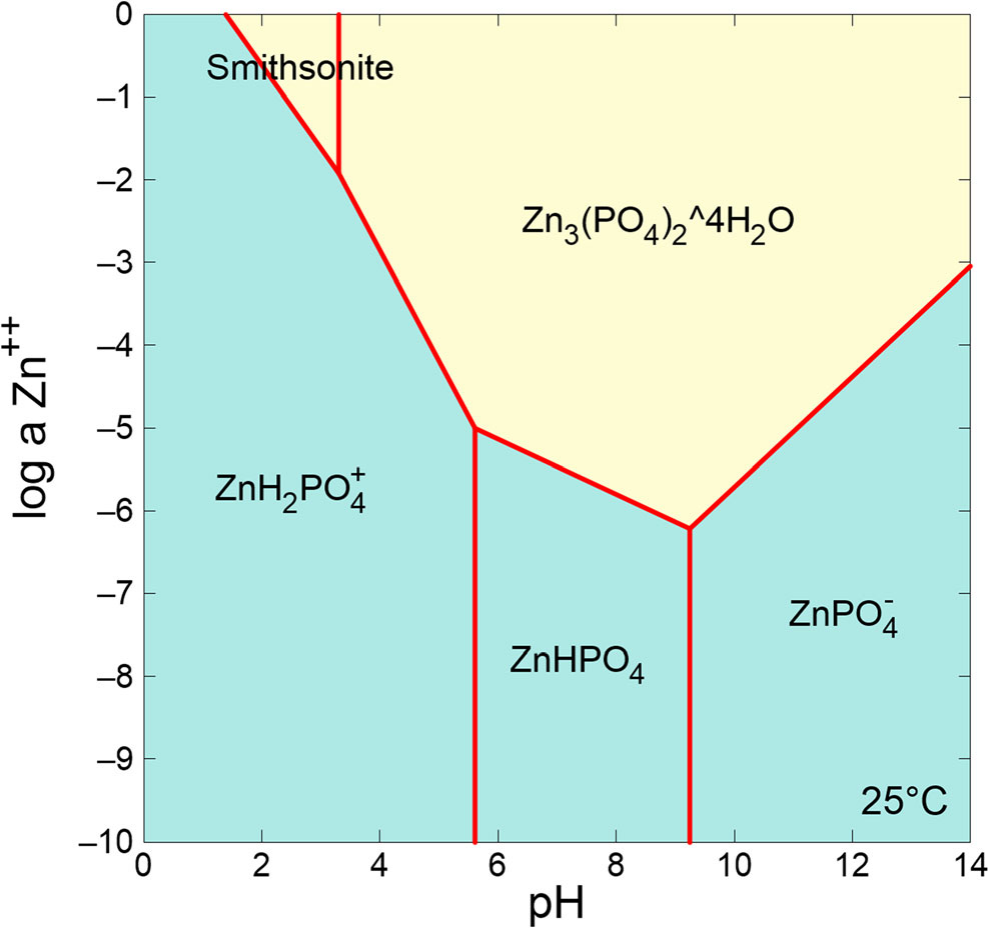
Geochemical simulation of Zn^2+^ versus pH in a simulated fungal system at 25 °C. The chemical parameters were set at 0.33-M CO_3_^2−^, 6.1-mM Cl^−^, 0.83-mM SO_4_^2−^, 0.66-M NH_4_^+^, 4-mM K^+^, 0.8-mM Mg^2+^, 1.7-mM Na^+^, 0.2-mM Ca^2+^, 0.02-mM Mn^2+^, and 9-μm Fe^3+^. The letter *a* on the *y*-axis represents the effective concentration of a given chemical species in the mixture (Li et al., unpublished data)

The pH is another crucial factor in determining the biodegradation of petroleum hydrocarbons and biotransformation of toxic metals. Changes in pH can alter fungal, and bacterial, community structure and enzyme activities as well as affecting metal speciation. Such effects of pH on the speciation of metal ions can be simulated using geochemical modeling software, e.g., Geochemists’ workbench (GWB) (Carrillo-Chávez et al. 2014; Li et al. 2019), MINEQL+ (Cloutier-Hurteau et al. 2007; Kocaoba 2020), and PHREEQC (Ceci et al. 2015; Liang et al. 2016b). For example, the speciation and solubility of Zn^2+^ in a simulated fungal system for metal remediation calculated using GWB showed that smithsonite (ZnCO_3_) (pH = 1.7–3.2) and Zn_3_(PO_4_)_2_∙4H_2_O (pH = 3.2–14) were the main mineral phases over different pH ranges (Fig. 3).

Temperature can influence the bioremediation of co-contaminated soil by affecting the chemistry of pollutants and fungal biodiversity (Rangel et al. 2018). The viscosity of petroleum increases at low temperatures and volatility is reduced which results in retardation of biodegradation. The highest degradation rates for hydrocarbon pollutants generally occur around 30–40 °C in the soil environment (Das and Chandran 2011). At higher temperatures, the solubility of PAHs and toxic metal ions increases which improves their bioavailability, although such high temperatures will also affect microbial community structure and activity. Compared with effects at 20 °C and 40 °C, 30 °C was found to be the optimum temperature for metal removal by *Beauveria bassiana* due to increased biomass production which provided more metal-binding sites (Gola et al. 2016).

### Importance of metabolites and enzymes

**Fig. 4:**
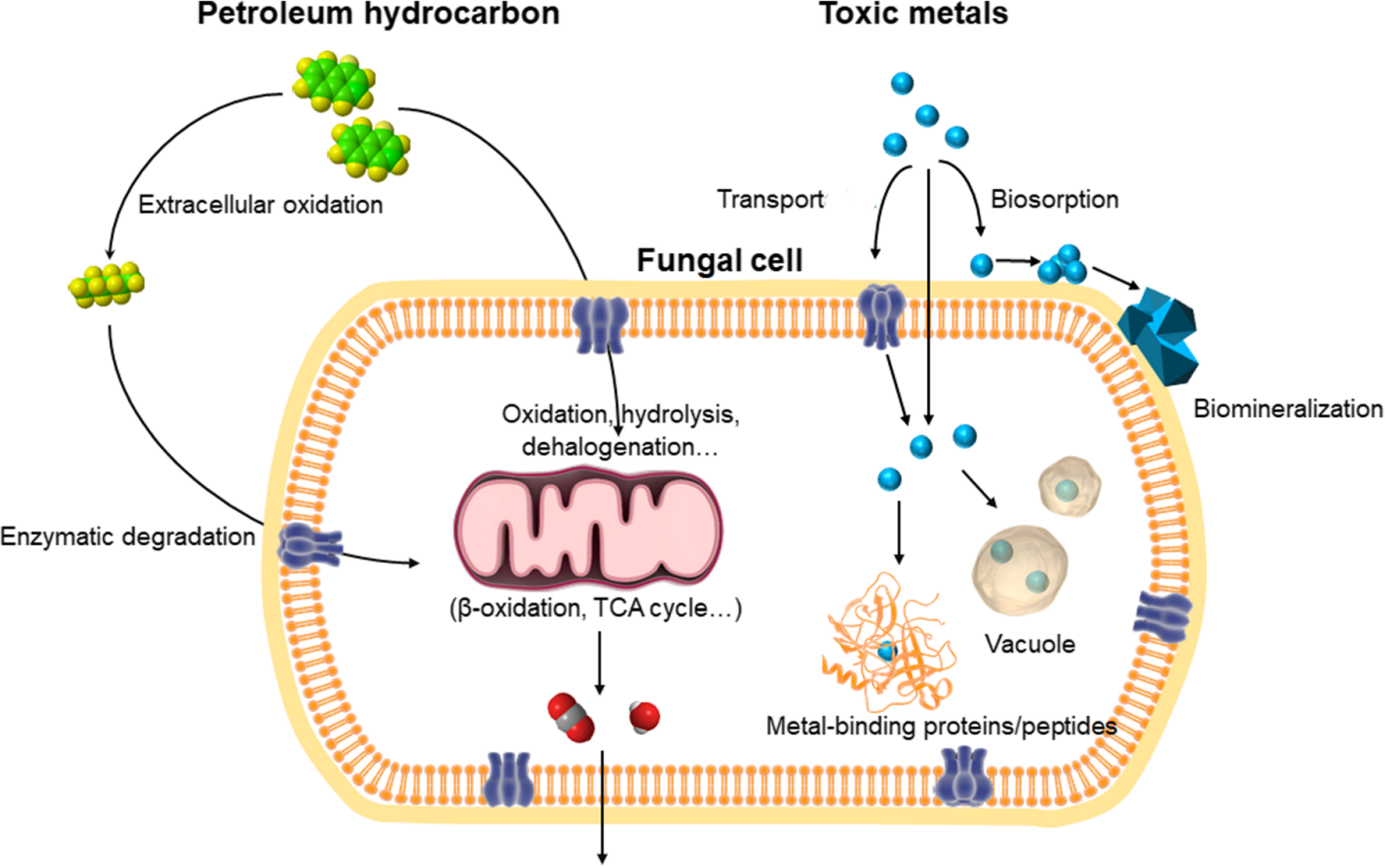
Simplified diagram of fungal interactions with petroleum hydrocarbons and toxic metals. Fungal cell membranes are permeable to petroleum hydrocarbons or simpler organic compounds oxidized by extracellular enzymes, which can undergo further metabolism including hydrolysis, dehalogenation, β-oxidation, and entry into the tricarboxylic acid cycle. Toxic metals can accumulate on fungal cell surfaces through biosorption, which can result in nucleation and subsequent precipitation of biominerals. Some metals can be intracellularly accumulated by active transport or diffusion through the cell membrane, and localized within vacuolar or other organellar compartments and/or be sequestered by sulfide, metal-binding proteins/peptides, and other macromolecules

Many transforming interactions between fungi and different pollutants depend on a variety of extracellular excreted substances and metabolites (Gadd 2004; Kirtzel et al. 2020). Fungi are capable of degrading petroleum hydrocarbons by secreting enzymes (e.g., laccases, tyrosinases, manganese peroxidases, cytochrome P450 monooxygenases, reductive dehalogenases), and affecting metal speciation by excretion of a variety of other metabolites (e.g., organic acids, amino acids, siderophores, extracellular proteins, etc.) (Fig. 4).

Petroleum hydrocarbons can be used by several fungal species as a carbon and energy source and assimilated into fungal biomass. Fungal taxa including *Amorphoteca*, *Neosartorya*, *Talaromyces*, *Aspergillus*, *Fusarium*, *Paecilomyces*, *Sporobolomyces*, *Cephalosporium*, *Penicillium*, and *Graphium* have all been reported to include potential degraders of petroleum hydrocarbons (Chulalaksananukul et al. 2006; Das and Chandran 2011; Varjani 2017). Some species can oxidize pollutants (e.g., phenols and aromatic amines) extracellularly by the production of laccases (Martínková et al. 2016), manganese peroxidases (Zhang et al. 2016), or lignin peroxidases (Falade et al. 2017; Grossart and Rojas-Jimenez 2016). Moreover, fungal cell membranes are permeable to many organic pollutants and these can be degraded by intracellular enzymes, e.g., cytochrome P450 (Ostrem Loss and Yu 2018), reductive dehalogenases (Stella et al. 2017), and nitroreductases (Tripathi et al. 2017; Xu and Zhou 2016), to simpler organic compounds, followed by further metabolism such as β-oxidation and entry into the tricarboxylic acid (TCA) cycle (Varjani 2017). Degradation of petroleum hydrocarbons consists of several different enzymatic steps with biodegradability depending on the chemical structure and other factors that affect fungal growth and metabolism, and pollutant chemistry and speciation. In general, biodegradation efficiency can be ranked as linear alkanes > branched alkanes > small aromatics > cyclic alkanes (Das and Chandran 2011; Guermouche M’rassi et al. 2015; Varjani 2017).

In co-contaminated soil, petroleum hydrocarbons may provide a carbon and energy source for certain fungal species while toxic metals can also exert significant effects on fungal populations and activity. Despite the potential toxicity of many metal species, many fungi can flourish in contaminated conditions although there may be shifts in species composition (Fomina et al. 2005; Gadd 2005; Gadd 2007). The major survival mechanisms involved can be explained as changes in toxic metal mobility resulting from various tolerance and resistance mechanisms (Gadd 2007; Gadd 2010). Fungi possess many mechanisms or properties that influence metal toxicity and mobility, including the production of metal-binding proteins, organic and inorganic precipitation, active transport, and intracellular compartmentalization, while cell walls and associated pigments and polysaccharides have significant metal-binding abilities (Gadd 1993; Gadd 2007). The secretion of fungal metabolites (e.g., metal-binding peptides, polysaccharides, amino acids, organic acids) is particularly important for metal and mineral transformations playing roles in both mobilization and immobilization of metal species (Gadd 2007; Gadd et al. 2014). Moreover, fungal phenolic polymers and melanin possess many potential metal-binding sites with oxygen-containing groups, such as carboxyl, phenolic and alcoholic hydroxyl, carbonyl, and methoxyl groups (Fomina and Gadd 2014). Fungal surface complex formation may be related to the coordination of metal ions with oxygen donor atoms and proton release (Gadd 2009):

$$ \mathrm{S}\hbox{-} \mathrm{OH}+{\mathrm{Cu}}^{2+}\rightleftharpoons \mathrm{S}\hbox{-} {\mathrm{OCu}}^{+}+{\mathrm{H}}^{+} $$

Bidentate surface complexation may also result:

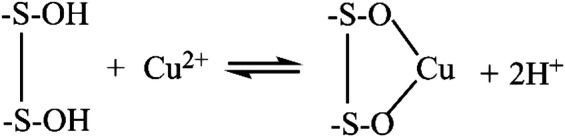



Metal immobilization appears particularly relevant to bioremediation approaches and fungi are capable of mediating precipitation of metals as insoluble oxalates, oxides, carbonates, and phosphates (Fomina et al. 2008; Gadd et al. 2014; Liang and Gadd 2017; Suyamud et al. 2020). For example, the liberation of phosphate from organic or inorganic phosphate hydrolysis proved to be an efficient method for metal immobilization, including Zn, Pb, La, and U, which were precipitated on and around hyphal surfaces (Ezawa and Saito 2017; Fomina et al. 2008; Liang et al. 2016a; Liang and Gadd 2017; Liang et al. 2015; Suyamud et al. 2020). Urease-positive fungi (e.g., *Neurospora crassa*, *Pestalotiopsis* sp., and *Myrothecium gramineum*) are promising candidates for the immobilization of toxic metals because the mechanism involved is associated with urea degradation. Such fungi grown in urea-containing medium hydrolyze urea producing ammonia and free carbonate which results in the precipitation of metals as carbonates, e.g., BaCO_3_, CdCO_3_, CoCO_3_, Cu_2_(OH)_2_CO_3_, La_2_(CO_3_)_3_, and NiCO_3_ (Li and Gadd 2017b; Li et al. 2019; Liang and Gadd 2017; Liu et al. 2019; Rautaray et al. 2004). Fungi can produce a variety of metal oxalates on interacting with metals and metal-bearing minerals including those of Ca, Cd, Co, Cu, Mg, Mn, Sr, Zn, Ni, and Pb (Gadd et al. 2014). Extracellular proteins, amino acids, and polysaccharides also play an important role in toxic metal immobilization. Extracellular nickel precipitation was associated with the removal of extracellular protein (Li et al. 2019), and it has been demonstrated that extracellular protein may act as a template for mineral formation, influencing the size of the resultant biominerals (Li and Gadd 2017a; Li et al. 2019; Liu et al. 2019).

### Future perspectives and conclusions

At present, bioremediation of soil co-contaminated with petroleum hydrocarbons and toxic metals is mostly focused on the removal, detoxification, or degradation efficiency of single or composite pollutants of each type. Little research has been carried out on the metabolism of fungi in response to complex pollutant stress. Fungal responses to petroleum hydrocarbons can be reflected by differences in metabolic responses, enzyme induction and synthesis, and extracellular metabolite production, which will also affect the migration and transformation of toxic metals. This is clearly a complex problem, affected by many variables, and sometimes limited by the availability of appropriate analytical techniques. For example, understanding the spatial distribution of toxic metals in the soil and/or the migration and transformation processes mediated by different fungal species depends on the sampling and analysis methods used. Sampling technology can be the main bottleneck that limits understanding due to heterogeneity of the soil in vertical and horizontal dimensions. In fact, toxic metals may show different gradient distributions over very small interfaces. Conventional techniques are also based on sampling and subsequent transport to the laboratory for analysis, but there may be subsequent changes during collection and storage due to, e.g., contamination and changes in environmental conditions (e.g., metabolic activity, pH, dissolved oxygen, Eh), which conceal the dynamic changes in biodiversity or chemical speciation that may occur in contaminated soil. To overcome these difficulties, new technology, including microbial metabolomics (Dombrowski et al. 2016; Tian et al. 2018) and proteomics, needs to be applied to the study of fungal bioremediation in co-contaminated soil, which could inform about metabolic responses under multiple pollutant conditions. This could provide scientific explanations for fungal responses to multiple contaminants at the molecular level (Aydin et al. 2017; Wang et al. 2017). Further, to obtain high-resolution spatial distribution characteristics of toxic metals and reveal interface reaction processes of soil-metals-fungal interactions, new methods such as gradient diffusion film technology (DGT) combined with laser ablation-inductively coupled plasma mass spectrometry could be applied to analyze dynamic changes in toxic metal speciation at soil-microbe interfaces (Challis et al. 2018; Feng et al. 2016; Koppel et al. 2020; Wang et al. 2018). Such approaches will further clarify fungal transformation mechanisms in soil contaminated with petroleum hydrocarbons and toxic metals and may contribute to more effective strategies for fungal bioremediation.
